# Intravenous Grafts Of Amniotic Fluid-Derived Stem Cells Induce Endogenous Cell Proliferation and Attenuate Behavioral Deficits in Ischemic Stroke Rats

**DOI:** 10.1371/journal.pone.0043779

**Published:** 2012-08-17

**Authors:** Naoki Tajiri, Sandra Acosta, Loren E. Glover, Paula C. Bickford, Alejandra Jacotte Simancas, Takao Yasuhara, Isao Date, Marianna A. Solomita, Ivana Antonucci, Liborio Stuppia, Yuji Kaneko, Cesar V. Borlongan

**Affiliations:** 1 Department of Neurosurgery and Brain Repair, Center of Excellence for Aging and Brain Repair, University of South Florida Morsani College of Medicine, Tampa, Florida, United States of America; 2 Departamento de Psicobiologia y Metodologia de las Cièncias de la Salud, Universidad Autónoma de Barcelona, Barcelona, Spain; 3 Department of Neurological Surgery, Okayama University Graduate School of Medicine, Dentistry and Pharmaceutical Sciences, Okayama, Japan; 4 Department of Biomedical Sciences, G. d'Annunzio University, Chieti-Pescara, Italy; 5 Department of Neuroscience and Imaging, School of Advanced Studies G.d'Annunzio, Chieti University and Stem TeCh Group, Aging Research Center, Chieti- Pescara, Italy; University of Queensland, Australia

## Abstract

We recently reported isolation of viable rat amniotic fluid-derived stem (AFS) cells [1]. Here, we tested the therapeutic benefits of AFS cells in a rodent model of ischemic stroke. Adult male Sprague-Dawley rats received a 60-minute middle cerebral artery occlusion (MCAo). Thirty-five days later, animals exhibiting significant motor deficits received intravenous transplants of rat AFS cells or vehicle. At days 60–63 post-MCAo, significant recovery of motor and cognitive function was seen in stroke animals transplanted with AFS cells compared to vehicle-infused stroke animals. Infarct volume, as revealed by hematoxylin and eosin (H&E) staining, was significantly reduced, coupled with significant increments in the cell proliferation marker, Ki67, and the neuronal marker, MAP2, in the dentate gyrus (DG) [2] and the subventricular zone (SVZ) of AFS cell-transplanted stroke animals compared to vehicle-infused stroke animals. A significantly higher number of double-labeled Ki67/MAP2-positive cells and a similar trend towards increased Ki67/MAP2 double-labeling were observed in the DG and SVZ of AFS cell-transplanted stroke animals, respectively, compared to vehicle-infused stroke animals. This study reports the therapeutic potential of AFS cell transplantation in stroke animals, possibly via enhancement of endogenous repair mechanisms.

## Introduction

Stroke is the fourth leading cause of death and the leading cause of disability in the United States [3]. To date, the only FDA-approved drug for ischemic stroke is tissue plasminogen activator (tPA). Due to the limited therapeutic window (4.5 hours from disease onset to tPA administration) and the risks associated with tPA (i.e., hemorrhagic transformation), only about 3 percent of ischemic stroke patients benefit from tPA therapy [4], [5]. In an effort to increase the therapeutic window, novel treatment strategies target a longer delay post-stroke, specifically the restorative phase which begins days to weeks post-stroke [1], [6], [7], [8].

Due to their ability to release anti-inflammatory cytokines that can potentially modify the hostile environment associated with the secondary cell death of the ischemic brain, stem cells have emerged as a potential therapeutic agent for stroke. The positive effects obtained by transplantation of AFS cells are ascribed to the grafted cells’ production of trophic factors and cytokines, as well as the increase in the levels of neurotrophic factors and reduced inflammatory response within the ischemic region in response to the administration of AFS cells. Additionally, the benefits of AFS cells may be attributable to its inhibition of apoptosis and oxidative stress, in tandem with stimulation of angiogenesis, neurogenesis, and synaptogenesis [1], [6], [7], [8]. Although stem cells can be isolated from many sources, including bone marrow, fetal and embryonic tissues, amnion-derived stem cells are an attractive source of stem cells because of many logistical and ethical advantages. Amnion-derived stem cells can be isolated from the tissue and the fluid [1], [6], [7], [8]. Harvesting these cells poses minimal risk of harming the fetus. Unlike amniotic tissue-derived stem cells, the AFS cells can be isolated from amniocentesis around 15–20 weeks gestation, whereas amniotic tissue-derived stem cells are harvested after childbirth. Extraction of AFS cells prior to delivery allows for the cells to be cultured, and in the event of childbirth-associated disorders (e.g., cerebral palsy) the stem cells can be amplified in advance and transplanted upon disease diagnosis within hours after birth. This efficient amplification process may be difficult with amniotic tissue-derived cells. AFS cells are isolated during an earlier phase of pregnancy, have a higher proliferative capacity, and their properties may more closely mimic embryonic stem cells compared to amniotic tissue-derived stem cells [1], [6], [7], [8]. Another advantage of AFS cells, compared to amniotic tissue-derived cells, is that the sterility of these cells is likely to be satisfied with AFS cells extracted via amniocentesis, but may be compromised when stem cells are harvested from the amnion tissue during child delivery.

**Figure 1:**
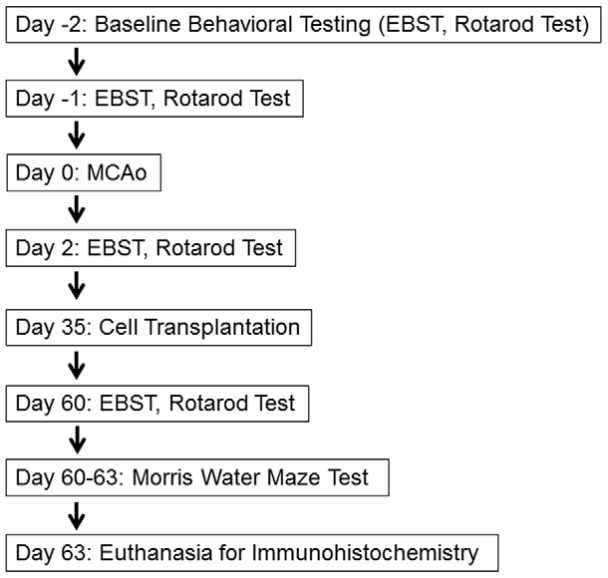
Experimental design is shown. Rats were subjected to a 60 minute transient MCAo and received intravenous transplants of AFS cells or vehicle. After behavioral evaluations, all rats were euthanized for immunohistochemical evaluations. EBST: elevated body swing test; MCAo: middle cerebral artery occlusion.

AFS cells can differentiate into multiple lineages [9], [10], [11], [12], [13], [14], [15]. Although the term “fluid” has been ascribed to AFS cells, cells isolated during amniocentesis contain a variety of stem cells originating from extra-embryonic and embryonic tissues [12]. The properties of AFS cells vary with gestational age [1], [6], [7], [8]. The versatility and plasticity properties of AFS cells fall somewhere in between the pluripotent embryonic stem cells and the multipotent adult stem cells [16], [17]. AFS cells have a high renewal capacity and can be expanded for over 250 doublings without any detectable loss of chromosomal telomere length [16]. The population doubling time for our AFS cells is approximately 30–36 hours. Taken together, these data provide support to the notion that the amniotic fluid is a rich and promising source of stem cells for clinical applications.

**Figure 2:**
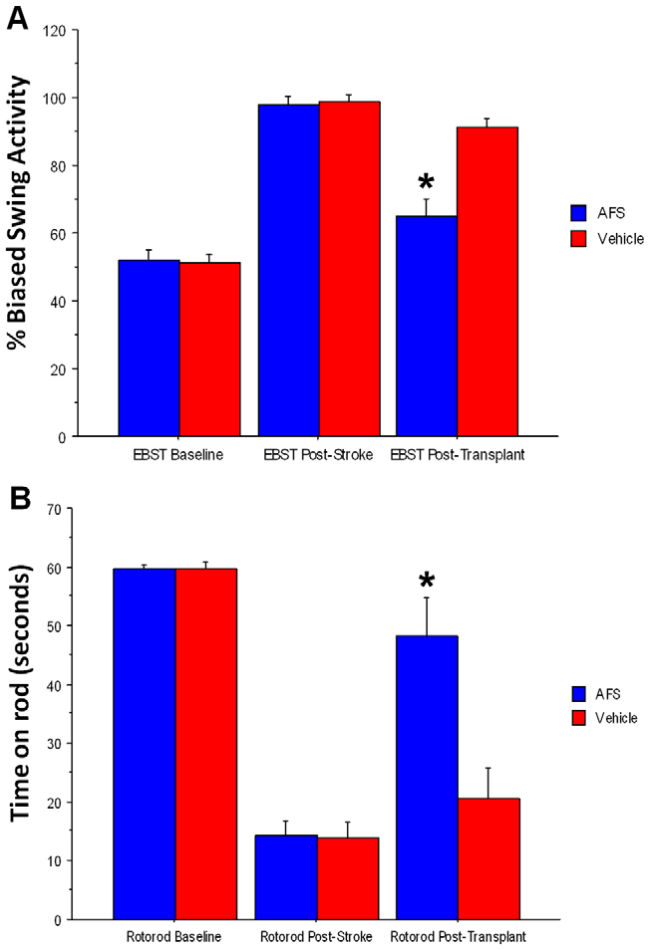
Motor deficits were ameliorated in the AFS cell-transplanted stroke animals. Results revealed that all animals exhibited no detectable bias in swing activity at baseline (p>0.05) (A) and all animals learned to balance on the rotating rod for 60 seconds (p>0.05) (B). EBST revealed a significant biased swing activity on day 2 post-MCAo. EBST conducted on day 60 post-stroke detected a significant decrease in swing bias in the AFS cell-transplanted stroke animals compared to the vehicle-infused stroke animals (*p<0.0001) (A). Rotarod test revealed significant deterioration in motor coordination on day 2 post-MCAo, but on day 60 post-MCAo revealed that the AFS cell-transplanted stroke animals exhibited significantly increased time spent balancing on the rotating rod compared to the vehicle-infused stroke animals (*p<0.0001) (B). Bars represent the mean ± SEM.

Transplantation of AFS cells has been explored in neurological disorders [1], [6], [7], [8]. Under standard neuronal induction protocols for stem cells, AFS cells possess preferential dopaminergic phenotypic commitment, making them a potentially valuable source of stem cells to treat Parkinson’s disease. In the same vein of specific fate commitment, AFS cells from second trimester amniotic fluid show the capacity to differentiate into all three germ layers and expressed Oct-4, Nanog, and SSEA-4 [18], which are pluripotent embryonic stem cell markers [1], [6], [7], [8]. These findings suggest that the amniotic fluid may be an attractive source of stem cells for neurological disorders.

**Figure 3:**
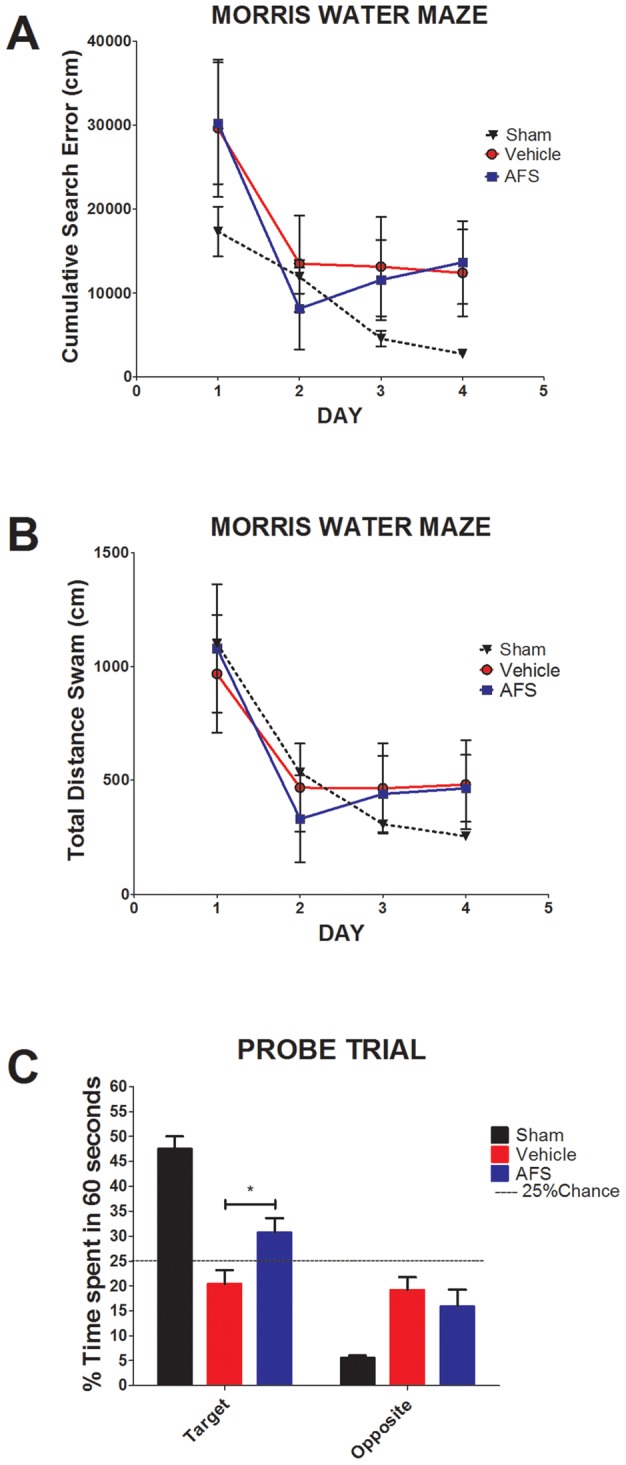
Reference memory, but not spatial navigation, is rescued by AFS cell transplantation. Results revealed that there were no significant differences in cumulative search error and total distance swam between the two treatment groups (A,B), indicating that spatial navigation was not improved in the AFS cell-transplanted stroke animals compared to the vehicle-infused stroke animals (p>0.05) (A). In contrast, reference memory was significantly improved in AFS cell-transplanted stroke animals compared to vehicle-infused stroke animals (C). Twenty-four hours after training in the MWM, rats were tested in a probe trial in which the platform was missing. Results revealed that AFS cell-transplanted stroke animals spent significantly more time in the target quadrant compared to vehicle-infused stroke animals (*p<0.05). Bars represent the mean ± SEM.

Transplantation of stem cells thus stands as a promising therapy for stroke; however, few studies focusing on AFS cells as a donor cell source for transplantation in stroke have been conducted. AFS cells delivered intracerebroventricularly in mice, three days after receiving a 60-minute middle cerebral artery occlusion (MCAo), attenuated short-term memory impairment and improved sensorimotor ability, somatosensory functions, and motor coordination seven days post-MCAo [19]. Although this study reports the beneficial effects of AFS cell transplantation in a mouse model of MCAo, histological analysis was not conducted and the animals were euthanized at an early time point following MCAo. Chronic stroke studies are needed to determine the long-term efficacy of AFS cell transplantation, as well as to elucidate the mechanism underlying their therapeutic effects. Here we analyzed the effects of intravenously transplanted AFS cells in MCAo animals using cognitive and motor tests and subsequent histological analysis of the brain for determination of therapeutic benefits and mechanism of action associated with this cell therapy for stroke.

**Figure 4:**
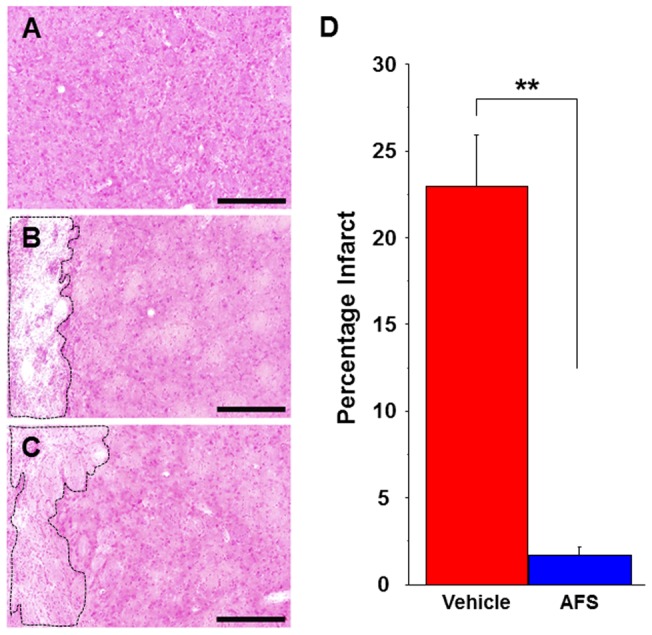
Infarct volume was reduced by AFS cell transplantation. Stronger H&E staining is found in the intact striatum (A) compared to the vehicle-infused stroke animals (B) and AFS cell-transplanted stroke animals (C). Infarct volume is significanly reduced in the AFS cell-transplated stroke animals (C) compared to vehicle-infused stroke animals (B). The striatum in the AFS cell-transplated stroke animals is clearly preserved compared to that of vehicle-infused stroke animals (A-D). Quantitative analyses revealed that percentages of the infarct volumes of rats receiving AFS cell transplants are significantly reduced (**p<0.01) (D). Data are shown as percentages of the infarct volumes present in the ipsilateral hemisphere relative to the contralateral hemisphere. Bars represent the mean ± SEM. Scale bars  = 200 µm. Black dotted box represents the infarct area.

## Materials and Methods

### Subjects

All experiments were conducted in accordance with the National Institute of Health Guide and Use of Laboratory Animals, and were approved by the Institutional Animal Care and Use committee of the University of South Florida, Morsani College of Medicine. Rats were housed two per cage in a temperature- and humidity-controlled room that was maintained on 12/12 hour-light/dark cycles. They had free access to food and water.

**Figure 5:**
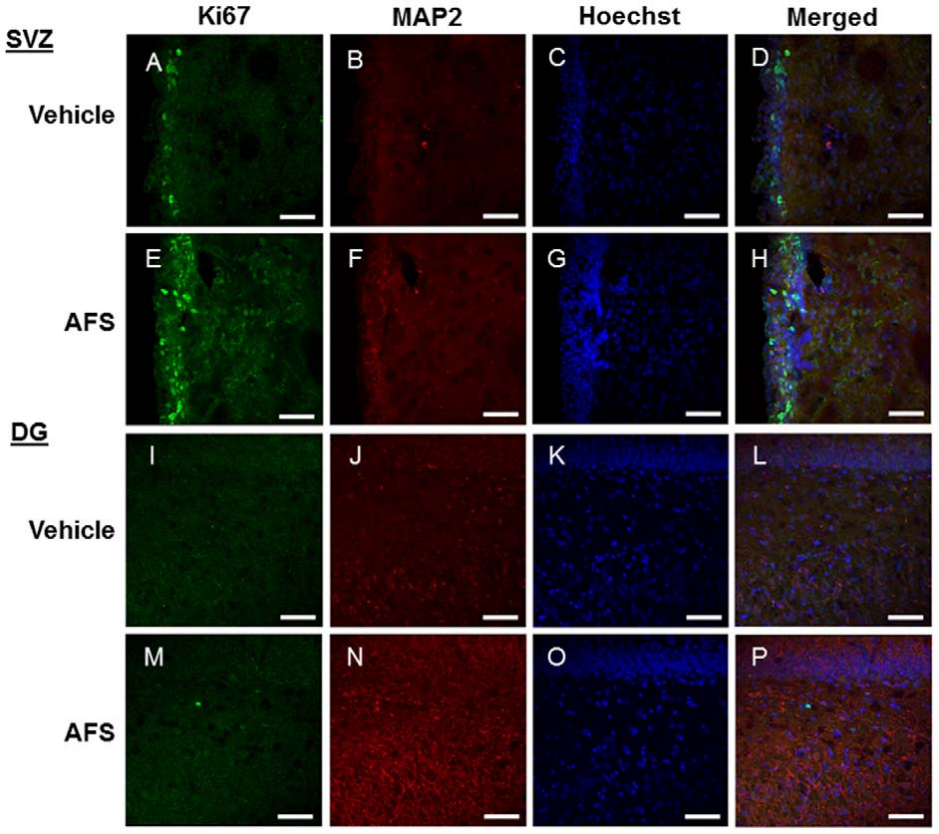
Enhanced endogenous cell proliferation and neuronal differentiation in the SVZ and DG following AFS cell transplantation. Ki67 (A, E, I, M) and MAP2 (B, F, J, N) staining revealed an apparent increase in the number of positive cells in the SVZ (A-H) and DG (I-P) of the AFS cell-transplanted stroke animals (E-H, M-P) compared to the vehicle-infused stroke animals (A-D, I-L). Ki67 and MAP-2 double-positive cells are shown in panels: D, H, L, and P. Green: Ki67, Red: MAP2, Blue: Hoechst. Scale bar  = 75 µm.

### Surgical procedures

Ten weeks old male Sprague-Dawley rats (n = 30) were subjected to stroke (n = 17) or sham surgery (n = 13) anesthetized by a mixture of 1–2% isoflurane in nitrous oxide/oxygen (69%/30%) via face mask. Body temperature was maintained at 37°C±0.3°C during the surgical procedures. The midline skin incision was made in the neck with subsequent exploration of the right common carotid artery (CCA), the external carotid artery, and internal carotid artery. A 4-0 monofilament nylon suture (27.0–28.0 mm) was advanced from the CCA bifurcation until it blocked the origin of the middle cerebral artery (MCA). Animals were allowed to recover from anesthesia during MCA occlusion (MCAo). After 60 minutes of transient MCAo, animals were re-anesthetized with 1–2% isoflurane in nitrous oxide/oxygen (69%/30%) using a face mask and reperfused by withdrawal of the nylon thread. Animals receiving the sham operation were anesthetized with 1–2% isoflurane in nitrous oxide/oxygen (69%/30%) via face mask. A midline incision was made in the neck and the right CCA was isolated. The animals were then closed and allowed to recover from anesthesia. We have standardized the MCAo model, with stroke animals showing at least 80% reduction in regional cerebral blood flow during the occlusion period as determined by laser Doppler (Perimed). To further ensure similar degree of stroke insults, physiological parameters including PaO2, PaCo2, and plasma pH measurements were monitored, and we found no significant differences in our stroke animals. Animals that did not display the 70% swing bias were excluded (n = 2). All animals were euthanized on day 63 post-MCAo for subsequent immunohistochemical investigations (n = 14). One animal died immediately after reperfusion, thus a motality rate of approximately 6% was observed post-MCAo in this study. The total number of animals in each group was as follows: n = 6 for the vehicle-infused stroke animals and n = 8 for the AFS cell-transplanted stroke animals. All data corresponding to the deceased animal and the animals excluded from the study based on a lack of swing bias were removed. A schematic diagram of experimental design is shown (Fig. 1).

**Figure 6:**
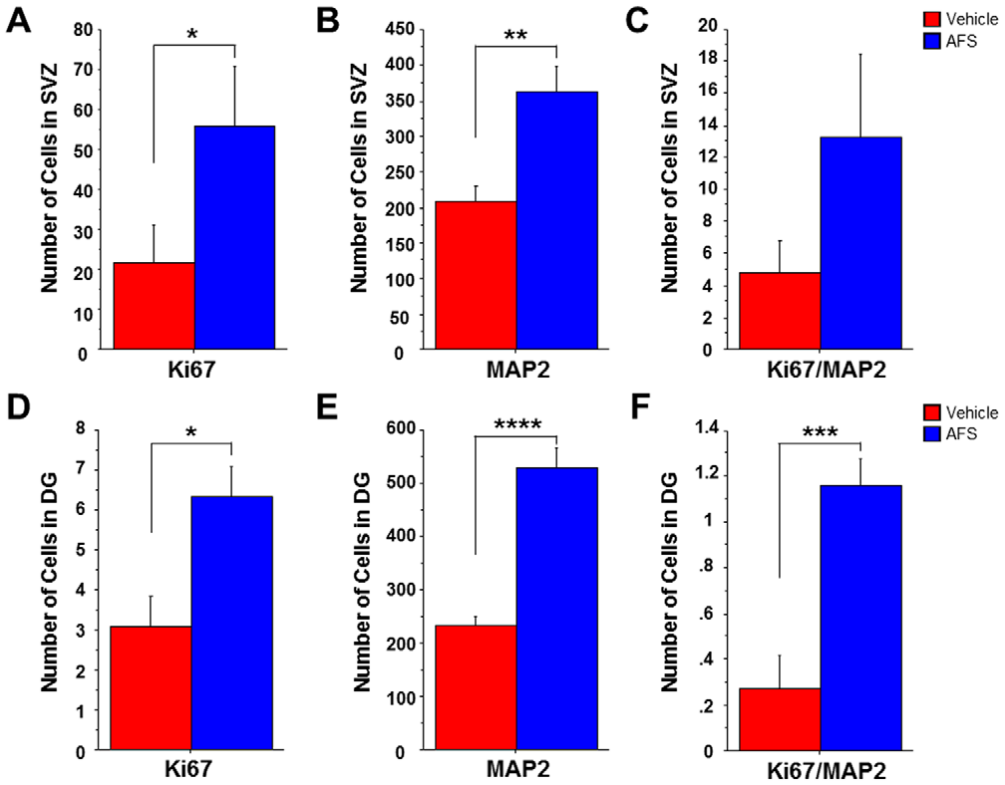
Quantification of cell proliferation and neuronal differentiation in the SVZ and DG. The number of cells labeled with Ki67 (A) and MAP2 (B) were significantly increased in the SVZ and DG of AFS cell-transplated stroke animals compared to vehicle-infused stroke animals (*p<0.05, **p<0.01, ****p<0.0001) (A, B, D, E). While there was a trend towards increased Ki67/MAP2 double-positive cells in the SVZ (p = 0.0946) (C), the number of cells labeled with both Ki67 and MAP2 were significantly increased in the DG of AFS cell-transplated stroke animals compared to vehicle-infused stroke animals (***p<0.001) (F). Bars represent the mean ± SEM.

### Isolation of AFS Cells

Amniotic fluid samples were obtained from timed pregnant Sprague-Dawley rats at gestation age 16–18 weeks. The study has been approved by the Ethics Committee for Biomedical Research of the "G. d'Annunzio" University, Chieti. For each sample, 2–3 ml of amniotic fluid, corresponding to a cell number ranging from 2×10^3^ to 2×10^6^ were centrifuged for 10 minutes at 1800 rpm. Pellets were resuspended in Iscove's modified Dulbecco's medium supplemented with 20% FBS, 100 U/ml penicillin, 100 μg/ml streptomycin (Sigma), 2 mM L-glutamine, 5 ng/ml basic fibroblast growth factor (FGF2) and incubated at 37°C with 5% humidified CO2. After 7 days, non-adherent cells were removed and the adherent cells allowed to growth in the same medium, which was changed every 4 days. When culture reached confluency (about 20 days after the primary culture), cells were treated with 0,05% trypsin and 0,02% EDTA, then counted and replaced in 25 cm^2^ culture flasks.

**Figure 7:**
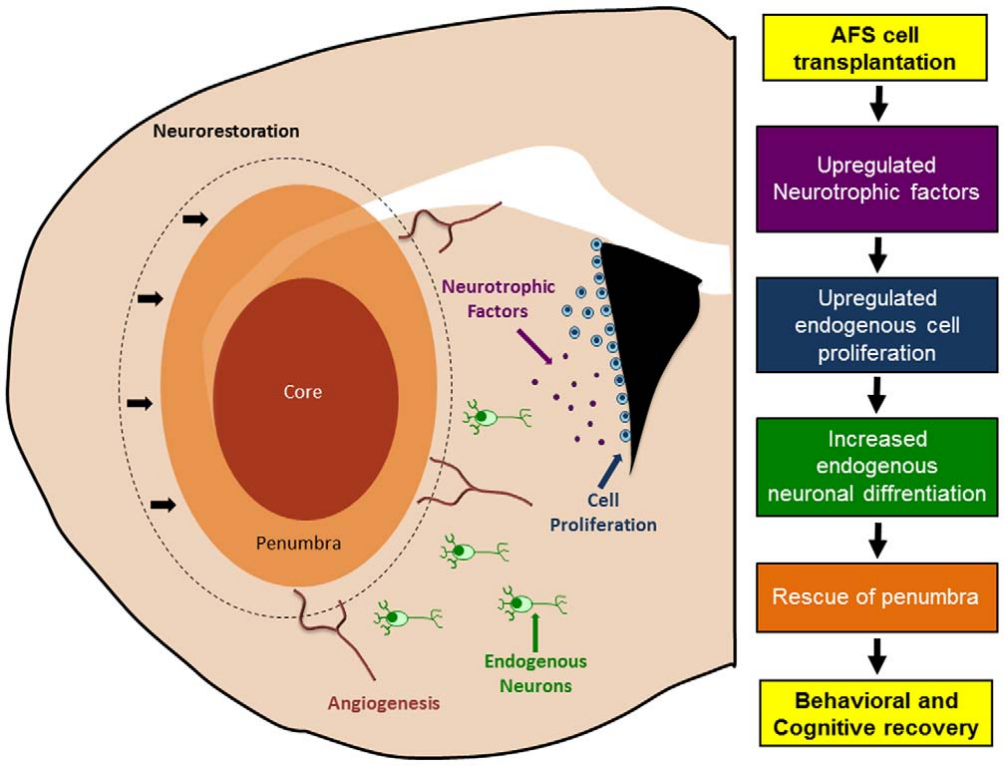
Schematic diagram of proposed mechanism of action of AFS cell transplants in stroke. This schematic diagram represents a speculative reparative mechanism underlying the functional recovery produced by AFS transplantation in stroke animals. Following AFS cell transplantation, neurotrophic factors are upregulated, which subsequently stimulates, endogenous cell proliferation, and neuronal differentiation to the injured area. These multi-pronged neurorestorative processes contribute to the rescue of the peri-infarct area leading to behavioral recovery.

### Intravenous administration of AFS cells or vehicle

On day 35 post-MCAo, rats were anesthetized with 1–2% isoflurane in nitrous oxide/oxygen (69%/30%) using a face mask. Transplantation of AFS cells (1 million viable cells in 1 ml of sterile saline; n = 8) or infusion of vehicle (equivalent volume of sterile saline; n = 6) or was performed intravenously via the jugular vein.

### Behavioral testing

Each rat was subjected to a series of behavioral tests to reveal motor, neurological, and cognitive performance of animals prior to MCAo, post-MCAo, and following transplantation. The tests included the elevated body swing test (EBST; motor test) and the rotarod test, both performed prior to and after MCAo, at days −1, 2 and 60 post-MCAo. The Morris Water Maze (MWM) test was performed on days 60, 61, 62, and 63 post-MCAo. EBST is a measure of asymmetrical motor behavior that does not require animal training or drug injection [20]. The rats were held, in the vertical axis, approximately 1 inch from the base of its tail and then elevated to an inch above the surface on which it has been resting. The frequency and direction of the swing behavior were recorded for over 20 tail elevations. A swing was counted when the head of the rat moved more than 10 degrees from the vertical axis to either side. Rats with unilateral ischemic stroke exhibited significant biased swing activity to the right (ipsilateral side), while those with no unilateral ischemic stroke displayed equal number of swings to the right and left. The total number of swings made to the biased side was divided by 20 to get percentages of the swings. For the criterion of successful MCAo model completion, biased swing behavior was set at 70% or higher. Rotarod test was also performed to evaluate the degree of hemiparesis and coordinated movements. Prior to MCAo surgery, all rats were pre-trained to stay on the accelerating rotarod (Accuscan, USA) at a constant speed of 8 rpm until they could remain on the rotarod for 100 seconds. After 3 consecutive days of pretraining, we performed three trials in which the rotational speed was gradually increased from 4 to 40 rpm within 5 minutes. The longest time the rats remained on the rotarod was measured as the baseline. Thereafter, the data at day 2 and day 60 post-MCAo were presented as percentages of the longest time on the rotarod of three trials (for each time point) relative to the baseline [21]. Finally, to reveal the cognitive effects of AFS cell transplants, the hippocampal dependent Morris water maze (MWM) task was used to evaluate the effects of AFS cell grafts on MCAo injured young rats (about 18 weeks old old and approximately 300 grams at this time of cognitive testing) on day 63 post-MCAo. All rats were placed in a tank that measured 1.5 meter in diameter with a 10 cm-diameter platform centered at 30 cm from the wall and submerged 1 cm below the surface of water. The water temperature was kept at 25°C. The performance on the MWM was measured over four days of training, with four trials per day. The platform was placed in any of four positions/quadrants: North, South, East, and West. Every animal had an assigned platform position, yet the starting zone (dropping zone) was randomly changed per trial. Once the animals found the escape platform, they were allowed to remain on the platform for 30 seconds between trials and then were transferred to a warm resting cage before the next trial. If any animal did not find its target platform within 60 seconds, the animal was guided to its target platform, and remained on the platfrom for 30 seconds. There was an approximate 30 minute delay between trials for the same rat. A probe trial, where the platform is missing, was evaluated to test consolidation of spatial memory of the two groups. The probe trial was conducted 24 hours after four days of acquisition of learning or training days. During the probe trial, the platform was taken out of the maze and every rat was allowed to freely swim for a period of 60 seconds. Using a computer tracking software (Noldus), the cumulative distance to target quadrant (also called cumulative search error), percent time spent in target quadrant, and the total distance swam, were assessed. The Morris water maze allows the investigator to analyze basic parameters such as place or spatial learning. The acquisition of learning is when the animal must learn how to use distal cues in order to navigate a path to find the hidden platform. During MWM testing, starting locations are randomly selected and they are different every time the animal is placed in the water [22], [23]. Also, it has been shown that MWM learning is not dependent on locomotor ability, because this does not affect swimming speed [23]. The cumulative search error shows the ability to learn the use of distal cues to find the platform. Also, it has been demonstrated that this type of measurement helps to distinguish spatial learning impairments from non-hippocampal dependent functions [22]. To asses learning ability, cumulative search error was calculated using the average distance to the target platform in meters multiplied by the time to the target. Also, total distance swam was calculated as a measure of learning. If animals fail to perform well in this test, it can be taken as learning disability and not as memory impairments. In our test, we showed that both groups of animals, namely vehicle-infused stroke animals and AFS cell-transplanted stroke animals, learned how to use the distal cues to find the hidden platform. Twenty-four hours after the acquisition of learning, a probe trial was given to all the animals to evaluate their consolidation of reference memory. The swimming performance and learning can further be dissociated during probe trials. During probe trial, memory measurements are insensitive to swimming speeds because only the percent time spent in target quadrant is calculated [22], [23]. Spatial retention or spatial memory was assessed during the probe trial by comparing the percent time spent in target platform to adjacent non-target quadrants [22].

### Brain tissue fixation and sectioning

Under deep anesthesia, rats were sacrificed on day 63 post-MCAo for immunohistochemical investigations (n = 6 for the vehicle-infused stroke animals and n = 8 for the AFS cell-transplanted stroke animals). Breifly, animals were perfused through the ascending aorta with 200 ml of cold phosphate buffered saline (PBS), followed by 200 ml of 4% paraformaldehyde (PFA) in phosphate buffer (PB). Brains were removed and post-fixed in the same fixative for 24 hours followed by 30% sucrose in PB until completely submerged. Six series of coronal sections were cut at a thickness of 40 μm with a cryostat and stored at −20°C.

### Immunohistochemistry

Every sixth 40 μm thick coronal tissue section beginning at AP −1.70 and ending at +0.20 relative to the bregma was isolated for morphological analysis of the SVZ [24]. Sections corresponding to 1.8 mm, 2.3 mm 2.8 mm, 3.3 mm, 3.8 mm, and 4.3 mm posterior to the bregma were randomly selected for quantitative analysis of the DG [24]. Free floating sections were washed three times for five minutes in PBS. For Ki67 and MAP2 staining, samples were blocked for 60 min at room temperature with 5% normal goat serum (Invitrogen) in PBS containing 0.1% Tween 20 (PBST) (Sigma). Sections were then incubated overnight at 4°C with rabbit polyclonal anti-Ki67 (1∶100; Abcam, ab15580) and mouse monoclonal anti-MAP2 (1∶500; Abcam, ab11267) with 5% normal goat serum. The sections were washed five times for ten minutes in PBST and then soaked in 5% normal goat serum in PBST containing corresponding secondary antibodies, goat anti-rabbit IgG-Alexa 488 (green) and goat anti-mouse IgG-Alexa 594 (red) (1∶500; Invitrogen), for 90 minutes. Finally, sections were washed five times for ten minutes in PBST and three times for five minutes in PBS, then processed for Hoechst 33258 (bisBenzimideH 33258 trihydrochloride, Sigma) for 30 min, washed in PBS, and cover-slipped with Fluoromount (Sigma). Control studies included exclusion of primary antibody substituted with 5% normal goat serum in PBS. No immunoreactivity was observed in these controls.

### Measurement of infarct volumes

A different set of serial sections corresponding to the same animal were stained with H&E for infarct volume calculations. Six coronal slices between the anterior edge and posterior edge of the infarct were collected and processed for hematoxylin and eosin (H&E) staining from each brain perfused at day 63 post-MCAo. Sections were cut at a thickness of 40 μm by a cryostat. Every sixth coronal tissue section, beginning at AP −1.70 and ending at AP +0.20 anterior to the bregma [24], were randomly selected for measurement of infarct. Brain sections were observed by a microscope equipped with a digital camera. The infarct volume of brain damage was measured in each slice and quantified by a computer assisted image analysis system (NIH Image Software, USA) and calculated by the following formula: [(area of the damaged region in each section) x 0.040] (mm^3^). Infarct volume was then expressed as a percentage of the ipsilateral hemisphere compared to the contralateral hemisphere.

### Statistical analysis

The data were evaluated statistically using analysis of variance (ANOVA) and subsequent post hoc Scheffe's or Bonferonni’s test for behavior. In addition, for analysis of the MWM data, cumulative search error during acquisition of learning was evaluated as a measure of spatial learning. The four trials per day were organized into blocks and analyzed using unpaired Student’s t-tests. Mann-Whitney's U test was used for immunohistochemical investigations. Statistical significance was preset at p<0.05.

## Results

### AFS cell transplantation ameliorates motor deficits caused by MCAo

Significant treatment effects were detected in motor behavior as revealed by repeated measures of ANOVA (F_1,12_ = 157.96, p<0.0001). EBST revealed that all animals exhibited no detectable bias in swing activity at baseline (p>0.05) (Fig. 2A), but displayed significant bias in swing activity at day 2 post-MCAo (p<0.0001) (Fig. 2A). EBST at day 60 post-MCAo revealed that the swing bias was significantly decreased in the AFS cell-transplanted stroke animals compared to the vehicle-infused stroke animals (p<0.0001) (Fig. 2A).

Similarly, significant treatment effects were detected in motor coordination as revealed by repeated measures of ANOVA (F_1,12_ = 48.19, p<0.0001). Rotarod testing conducted prior to MCAo showed there was no significant difference between the two groups of animals, in that all animals learned to balance on the rotating rod for about 60 seconds (p>0.05) (Fig. 2B). However, at day 2 post-MCAo, rotarod testing revealed a significant decrease in the time spent balancing on the rotating rod in both groups (p<0.0001) (Fig. 2B). Rotarod testing conducted at day 60 post-MCAo revealed that the AFS cell-transplanted stroke animals exhibited significantly increased time spent balancing on the rotating rod compared to the vehicle-infused stroke animals (p<0.0001) (Fig. 2B).

### AFS cell transplantation attenuates cognitive impairments caused by MCAo

Both groups of rats were tested for spatial memory using a standard MWM design. In this behavioral paradigm, the rats were trained for four trials a day for four consecutive days to find the hidden escape platform. During the MWM task, there were no differences in performance during acquisition of learning task (p>0.05) (Fig. 3A, B). Additionally, there were no disparities in terms of motor function such as swimming capabilities, indicating they equally escaped from the water by finding the platform during the 60 second learning trials. There are four quadrants in the morris water maze, chance performance is equal to 25%. In the probe trial (Fig. 3C) a dashed line has been added at 25% to represent chance [25], [26]. During the probe trial, ANOVA revealed significant treatment effects (F_1,12_ = 7.586, p<0.001) with a posthoc pairwise test detecting significantly improved performance of AFS cell-transplanted stroke animals compared to vehicle-infused stroke animals in reference memory (Bonferroni’s test, p<0.05) (Fig. 3). We routinely maintain a sham control group as a reference condition on which to assess the experimental treatment groups. This sham group was age-matched, the same gender, and the same strain as the experimental treatment groups, and subjected to the same surgical maneuvers except for the major operation, which in this case was the stroke insult. In addition, our laboratory and others have historically shown the performance of young sham animals in both cumulative search error and distance swam. Sham data were provided as a reference control group (n = 20) for the present vehicle-infused stroke animals and AFS cell-transplanted stroke animals in order to show that MCAo had an impact on cognitive function [22], [23], [27]–[31].

### AFS cell transplantation decreases infarct volume caused by MCAo

H&E staining revealed that the AFS cell-transplanted stroke animals exhibited significantly decreased infarct volumes compared to the vehicle-infused stroke animals (Fig. 4A, B, and C). There was approximately a 21% difference between the infarct volumes of the AFS cell-transplanted stroke animals and the vehicle-infused stroke animals, which equated to about a 92% reduction in infarct volume in the AFS cell-transplanted stroke animals (p<0.01) (Fig. 4D). This reduction in infarct volume may mediate the improved motor activity, motor coordindation, and memory performance.

### AFS cell transplantation after MCAo increases cell proliferation and decreases neuron loss in the sub-ventricular zone

Immunofluorescence revealed that the number of cells labeled with Ki67, a cell proliferation marker, was significantly upregulated in the subventrical zone (SVZ) of the AFS cell-transplanted stroke animals compared to the vehicle-infused stroke animals (p<0.05) (Fig. 5, 6A). Similarly, the number of MAP2 positive cells was significantly increased in AFS cell-transplanted stroke animals compared to the vehicle-infused stroke animals (p<0.01) (Fig. 5, 6B). A trend towards increased Ki67/MAP2-positive cells was observed in the SVZ of AFS cell-transplanted stroke animals compared to vehicle-infused stroke animals (p = 0.0946) (Fig. 5, 6C).

### AFS cell transplantation after MCAo increases cell proliferation and decreases neuron loss in the dentate gyrus

The number of cells labeled with Ki67 was significantly increased in the dentate gyrus of the AFS cell-transplanted stroke animals compared to vehicle-infused stroke animals (p<0.05) (Fig 5, 6D). MAP2 was also significantly increased in the DG of the AFS cell-transplanted stroke animals compared to vehicle-infused stroke animals (p<0.0001) (Fig. 5, 6E). Similarly, the number of cells labeled with Ki67/MAP2 double-positive cells was significantly increased in the DG of AFS cell-transplanted stroke animals compared to vehicle-infused stroke animals (p<0.001) (Fig. 5, 6F).

## Discussion

This study reports the therapeutic potential of AFS cell transplantation in stroke animals, characterized by attenuation of stroke-induced behavioral and histological deficits, possibly via enhancement of endogenous repair mechanisms. We demonstrate that intravenous AFS cell transplantation ameliorated both motor and cognitive impairment and was accompanied by reduction of infarct volume. These functional improvements in AFS cell-transplanted stroke animals coincided with increased cell proliferation and neuronal differentiation in the two neurogenic sites: SVZ and DG, suggesting the role of graft-induced host tissue repair in this brain remodeling process following stroke (Fig. 7).

In the clinic, stroke symptoms involve deficits in sensorimotor and cognitive functions [32], [33], [34]. To date, most stroke animal models have focused on motor impairments, neglecting the cognitive declines that proceed after the brain insult [35], [36], [37]. Studies on the pathology of ischemic stroke support the negative impact that this insult imposes to hippocampal functions. It is well known that learning and memory consolidation are part of brain cognitive functions. The hippocampus plays a pivotal role when encoding and retaining new memories [38]. Animal models of hippocampal ablation have helped to address the function of this brain structure, and its relationship with learning and memory formation [39], [40], [41]. Adult neurogenesis or the generation of new neurons as the brain matures, and its decline during aging correlates with the deterioration of cognitive functions [42], [43], [44]. The post-stroke neuropathological cascade of events affecting the hippocampal area implicates the down regulation of glutamate receptors, decreased neurogenesis, exacerbated glial activation and chronic inflammation [45], [46], [47]. In addition, there are in vivo stroke studies characterizing hippocampus-mediated cognitive impairments clearly delineating these ischemia-induced learning and memory deficits from sensorimotor and neurological dysfunctions [45], [47], [48].

Consequently, the present study expands experimental techniques to assess both motor and cognitive symptoms in an animal model of stroke to fully characterize the disease progression, as well as the therapeutic benefits of AFS cell transplantation. Our present observations on cognitive testing showed no difference in learning performance between the two treatment groups during MWM training, but the AFS cell-transplanted stroke animals exhibited a significantly improved reference memory during the MWM probe trial compared to the vehicle-infused stroke animals, which correlated with our results showing significant increase of neurogenesis relative to vehicle-infused stroke animals in the DG of the hippocampus of AFS cell-transplated stroke animals. In particular, the AFS cell-transplanted stroke animals spent a significantly longer time exploring the target quadrant than the vehicle-infused stroke animals, but both groups spent a comparable amount of time of exploration inside the non-target/opposite quadrant. This increment in exploration time in target quadrant indicates that the AFS cell-transplanted stroke animals displayed a recovery of hippocampal dependent reference memory in that they discriminated between target and non-target quadrants. On the other hand, the vehicle-infused stroke animals exhibited a selective reference memory deficit in that they could not discriminate between target and non-target quandrants. Our data support the MWM test as a hippocampal dependent task for spatial memory and reference memory [49], [50], [51], [52], and long-term potentiation [2], [53], [54], [55]. These data allow us to advance the concept that transplanted AFS cells selectively aid in the recovery of the reference memory, but not task acquisition. The differences in reference memory between groups are not attributable to motor deficits or swimming abilities, as both groups showed a similar performance during neurological and motor testing (EBST and Rotarod), and during MWM training. Cognitive improvements of the transplanted group were also previously associated with the reduction of infarct volumes of the striatum [56], [57], [58], [59], a brain structure implicated in the cognitive functions of memory systems [60], [61], [62]. Taken together, these results reveal that transplanted AFS cells reduced the infarction of brain areas implicated in the performance of cognitive tasks, and enhanced the level of neurogenesis when compared to our vehicle-infused stroke animals.

AFS cells transplanted intracerebroventricularly three days post-MCAo attenuate cognitive deficits associated with stroke [19]. To date, stroke studies utilizing AFS cells have focused on the intracerebral cell transplantation route [19], [63]. A recent study examined the feasibility of intraperitoneal administration of AFS cells in newborn rats [64]. The present intravenous route of transplantation is a minimally invasive procedure and poses less risk to the patient compared to intracerebral transplantation. Due to the acute nature of stroke, a peripheral injection route is preferred so that therapeutics can be administered quickly after the onset of a stroke. Here, AFS cells were transplanted intravenously, enhancing the clinical relevance of this study. A clinical trial, from Celgene Cellular Therapeutics, that utilizes placenta/amnion-derived stem cells transplanted intravenously in stroke patients is currently underway [65].

The present observations of transplant-mediated recovery of motor and cognitive functions mirror the findings by Rehni and colleagues [19]. However, the present study expanded the short timeline of seven days in that previous study to 63 days. The guidelines of Stem cell Therapeutics as an Emerging Paradigm for Stroke (STEPS) require that stem cell therapies be tested in multiple strains of adult and aged male and female rats to evaluate safety and efficacy [35], [36], [37]. The STEPS criteria also emphasize the need for long term (at least one month) testing after the administration of neurorestorative therapies [35], [36], [37]. Long term testing is critical for evaluating the safety of stem cell administration, as well as determining the long-term efficacy of transplantation. A novel motor test called the rotarod slip test, which monitors the number of slips of the paralytic hind limb from a rotarod, is able to detect mild hindlimb paresis in the acute and sub-acute phase after [66]. We will incorporate this new rotorod slip test in future studies to characterize functional deficits in stroke, as well as transplant-mediated therapeutic benefits. The present behavioral tests have been accepted by the FDA as appropriate tests for translating stem cell products to the clinic.

The present study transplanted rat AFS cells into stroke rats, which supports the envisioned use of allogenic grafts in the clinic. The differentiation potential of AFS cells falls somewhere between the pluripotent embryonic stem cells and the multipotent adult stem cells, making the amniotic fluid an optimal source of stem cells [1], [12]. Furthermore, since AFS cells can be obtained during a routine amniocentesis isolation of AFS cells does not harm the developing fetus [1], [67]. Our lab has a long-standing interest in intravenous stem cell transplantation for neurological disorders, in particular stroke [68], [69], [70], [71] and the use of amniotic tissue and fluid [1], [6], [7], [8].

Histologic analysis revealed that AFS cell-transplanted stroke animals exhibited a reduction in infarct areas compared to the vehicle-infused stroke animals. his robust reduction in cerebral infarcts was likely due to transplanted AFS cells increasing cell proliferation in tandem with a decrease in neuronal loss in both the SVZ and the hippocampus [2]. As the two neurogenic niches in the brain, the SVZ and DG, are critical to the repair of damaged brain tissue [72], [73], [74], [75]. The results suggest that AFS cell transplantation might have boosted endogenous repair mechanisms by maximizing the potential of these two neurogenic sites to confer a host brain remodeling process. The lack of labeled AFS cells is a limitation to this study, which prevented examination of the fate of the transplanted cells. Such cell labeling process (e.g., viral vector or DiL dye labeling) may change the cell phenotype and alter the cell’s functional capabilities [76], [77], [78]. With the present demonstration of functional effects of the transplanted AFS cells, future studies will evaluate the similar therapeutic benefits, as well as fate of transplanted labeled and non-labeled AFS cells.

In summary, intracerebral administration of AFS cells ameliorate in the short-term the behavioral deficits associated with stroke [19]. That AFS cells when transplanted intravenously on day 35 similarly attenuated motor and cognitive impairments accompanied by reduced histological deficits and increased cell proliferation and differentiation in the host brain (Fig. 7) represent highly innovative observations. These findings directly advance our basic scientific knowledge about a potent mechanism of brain repair in stroke, and provide pivotal guidance into the translational applications of cell therapy to stroke patients.
